# Growth Rate of and Gene Expression in *Bradyrhizobium diazoefficiens* USDA110 due to a Mutation in blr7984, a TetR Family Transcriptional Regulator Gene

**DOI:** 10.1264/jsme2.ME16056

**Published:** 2016-07-05

**Authors:** Naoko Ohkama-Ohtsu, Haruna Honma, Mariko Nakagome, Maki Nagata, Hiroko Yamaya-Ito, Yoshiaki Sano, Norina Hiraoka, Takaaki Ikemi, Akihiro Suzuki, Shin Okazaki, Kiwamu Minamisawa, Tadashi Yokoyama

**Affiliations:** 1 Institute of Agriculture, Tokyo University of Agriculture and Technology; 2 Graduate school of Agriculture, Tokyo University of Agriculture and Technology; 3 Department of Agricultural Sciences, Faculty of Agriculture, Saga University; 4 Graduate schools of Bioresource Production Science, Nihon University; 5 Faculty of Agriculture, Tokyo University of Agriculture and Technology; 6 Graduate School of Life Sciences, Tohoku University

**Keywords:** *Bradyrhizobium diazoefficiens*, cell division, glutathione *S*-transferase

## Abstract

Previous transcriptome analyses have suggested that a gene cluster including a transcriptional regulator (blr7984) of the tetracycline repressor family was markedly down-regulated in symbiosis. Since blr7984 is annotated to be the transcriptional repressor, we hypothesized that it is involved in the repression of genes in the genomic cluster including blr7984 in symbiotic bacteroids. In order to examine the function and involvement of the blr7984 gene in differentiation into bacteroids, we compared the free-living growth/symbiotic phenotype and gene expression between a blr7984-knockout mutant and the wild-type strain of *Bradyrhizobium diazoefficiens* USDA110. The mutant transiently increased the cell growth rate under free-living conditions and nodule numbers over those with the wild-type strain USDA110. The expression of three genes adjacent to the disrupted blr7984 gene was strongly up-regulated in the mutant in free-living and symbiotic cells. The mutant also induced the expression of genes for glutathione *S*-transferase, cytochrome *c* oxidases, ABC transporters, PTS sugar transport systems, and flagella synthesis under free-living conditions. bll7983 encoding glutathione *S*-transferase was up-regulated the most by the blr7984 disruption. Since redox regulation by glutathione is known to be involved in cell division in prokaryotes and eukaryotes, the strong expression of glutathione *S*-transferase encoded by the bll7983 gene may have caused redox changes in mutant cells, which resulted in higher rates of cell division.

Rhizobia, which are soil microorganisms, form root nodules after they infect the roots of legume plants and fix nitrogen into ammonium in these nodules, which is then provided to the host plants as a nitrogen source. In the process of symbiosis, rhizobia differentiate into bacteroids, which is a morphology specific to symbiosis. The expression of nitrogenase enzymes encoded by *nif* genes is induced in bacteroids and cell division is suppressed (14). In root nodules, bacteroids are surrounded by peribacteroid solution (PBS), which is enclosed within peribacteroid membranes derived from the plasma membranes of host plant cells (25). PBS contains active enzymes such as α-glucosidase, malate dehydrogenase, glutamate oxalacetate transaminase (12), and α-mannosidase II (11). Tejima *et al.* (23) examined the composition of amino acids, organic acids, and sugars in PBS purified from soybean root nodules, and found that it contains abundant amounts of sugars and low levels of amino acids. The sugar composition of PBS was predominated by inositols, particularly *myo*-inositols.

Since PBS is metabolically active and has a specific composition of metabolites, it is considered to contain substances that induce the differentiation of rhizobia into bacteroids. In order to confirm this, we compared the genome-wide expression profile of *Bradyrhizobium diazoefficiens* USDA110 cells cultured in purified PBS from soybean root nodules and bacteroids isolated from soybean root nodules using macroarrays. We found that the PBS treatment preferentially induced genomic regions in a large symbiosis island including various symbiotic genes such as *nod*, *fix*, *nol*, and *noe*. In the symbiosis island, 75% of regions were commonly induced in bacteroids, while general repression outside of the symbiosis island in bacteroids was not observed in PBS-treated cells. These results suggest that PBS contains some, but not all substances that induce the expression of genes involved in differentiation into bacteroids (16).

In the macroarray analysis, we detected a genomic cluster region from coordinated 8763900 to 8786820 relative to the replication origin as 1, in which all 9 clones were repressed in bacteroids and most were also repressed by the PBS treatment (Supplementary Table S1 in [16]). Among the clones repressed in bacteroids and by the PBS treatment, the brb05976 clone contained the gene, blr7984, annotated as a transcriptional regulatory protein tetracycline repressor (TetR) family. TetR family proteins bind to the tetracycline response element in promoters and repress the transcription of genes in the absence of tetracycline, whereas TetR proteins leave the promoter in its presence and RNA polymerase then initiates transcription (6). Since blr7984 is annotated to be a transcriptional repressor, we hypothesized that it is involved in the repression of genes in the genomic cluster in bacteroids.

In order to analyze the function of blr7984, we generated a knockout mutant of this gene, and examined the phenotypes of the mutant in free-living and bacteroid statuses.

## Materials and Methods

### Generation of the blr7984 knockout mutant

The brp00457 clone, in which the genome region (8761124–8770880) of *B. diazoefficiens* USDA110 is subcloned in pUC18, was digested with *EcoR*I and *Bgl*II. The 1-kb fragment containing the blr7984 gene obtained was subcloned in pK18mob (19) to produce pK18mob-blr7984. pK18mob-blr7984 contained a single *Sma*I site in the region derived from the blr7984 sequence. The Ω cassette encoding genes for resistance to spectinomycin and streptomycin obtained by the digestion of pHP45Ω (18) with *Sma*I was subcloned in the *Sma*I site of pK18mob-blr7984 to produce pK18mob-blr7984Ω. The plasmid pK18mob-blr7984Ω was introduced into *B. diazoefficiens* USDA110 by triparental mating, with pRK2013 as a helper plasmid (5). The double cross-over mutant, in which the Ω cassette was inserted into the blr7984 region of the genome of *B. diazoefficiens* USDA110, was selected on yeast extract mannitol medium solidified with agar (YM, [8]) containing polymixin 50 mg L^−1^, spectinomycin 50 mg L^−1^, and streptomycin 50 mg L^−1^. The insertion of the Ω cassette in blr7984 was confirmed by PCR with primers specific to the blr7984 gene (blr7984-F: 5′-GACATTTATAGCCT TCAAAACCCG-3′ and blr7984-R: 5′-GAGCGAGAGCAGAGTC AGGAAT-3′) and to the Ω cassette (OMEGA-F: 5′-TGATTTGC TGGTTACGGTGA-3′ and OMEGA-R: 5′-GCCAAGCGATCTTC TTCTTG-3′).

### Growth rate of the *B. diazoefficiens* wild-type and Δblr7984 mutant

A single colony of the *B. diazoefficiens* USDA110 wild-type or Δblr7984 mutant was pre-cultured in YM broth medium (YMB, [8]) for 4 d. One milliliter of pre-cultured cells adjusted to 10^8^ cells mL^−1^ were transferred into 100 mL of YMB for further growth at 28°C. Cells were sampled at intervals and cell numbers were counted using the plate dilution method.

### Nodulation and nitrogen fixation by the *B. diazoefficiens* wild-type or Δblr7984 mutant

One hundred and twenty grams of vermiculite washed with deionized water and dried at 120°C for 2 day was packed in glass containers with a diameter of 5 cm and height of 12 cm. The moisture content of vermiculite was adjusted to 0.6 m^3^ m^−3^ of the maximum water-holding capacity by the addition of an N-free nutrient solution (23). The containers with vermiculite were covered with aluminum foil and sterilized by autoclaving at 121°C for 20 min. Soybean (*Glycine max* L. cv. Enrei) seeds were surface-sterilized with a diluted sodium hypochlorite solution (0.01 kg kg^−1^ available chlorine), washed with sterilized water, sown into vermiculite, and placed in a plant growth chamber (day 24°C for 16 h/night 16°C for 8 h). Moisture in the vermiculite bed was maintained at approximately 0.6 m^3^ m^−3^ of the maximum water-holding capacity by supplementing with sterilized water. One week after germination, soybean plants were inoculated with the wild-type or Δblr7984 mutant of *B. diazoefficiens* USDA110 by adding cells suspended with sterilized water at 10^8^ cells mL^−1^ to vermiculite. Plants were sampled 3, 5, and 9 weeks after germination.

Roots were gently washed with tap water and the numbers and fresh weights of visible root nodules were examined. The nitrogen fixation activities of root nodules were investigated by measuring acetylene reducing activity. Root nodules were placed in a 300-mL tightly capped glass bottle. Thirty milliliters of air was pulled out with a syringe and the same volume of acetylene was injected. After the incubation of root nodules at 28°C for 1 h, 1 mL of gas was pulled out from the bottle and the concentration of ethylene in it was measured by GAS CHROMATOGRAPH GC-2014 (SHIMADZU, Kyoto, Japan). A column (diameter of 3 mm and length of 1 m) packed with porapak N (80–100 mesh) was used for separation at 50°C. Nitrogen gas was flushed through the column at 50 mL min^−1^ as a carrier.

### Microarray analysis

A single colony of the wild-type or Δblr7984 mutant of *B. diazoefficiens* USDA110 was pre-cultured in YMB for 4 day. One milliliter of pre-cultured cells, adjusted to 10^8^ cells per mL, was diluted in 100 mL of YMB and cultured for 36 h at 28°C. Cells pelleted by centrifugation at 4°C were washed twice with ice-cold phosphate-buffered saline (8 g of NaCl, 0.45 g of NaH_2_PO_4_·2H_2_O, 3.225 g of Na_2_HPO_4_·12H_2_O in 1 L), and total RNAs were then isolated from them using RNAiso Plus (Takara Bio, Shiga, Japan). After being treated with DNase I (Thermo Fisher, Waltham, MA, USA), total RNAs were cleaned using an RNeasy Mini Kit (Qiagen, Hilden, Germany). Cleaned total RNAs were subjected to a microarray analysis using the Gene Atlas system (Affymetrix, Santa Clara, CA, USA) with an Affymetrics Soybean Gene 1.1 ST Array Strip (includes *B. diazoefficiens*). PartekExpress (Ryoka systems, Tokyo, Japan) was used to analyze the results obtained from microarray experiments and data for *B. diazoefficiens* genes were compared between the wild-type and Δblr7984 mutant. Microarray experiments were performed with three biological replicates and the results obtained were statistically analyzed with ANOVA. Annotations of the selected genes were confirmed with Rhizobase (http://genome.microbedb.jp/rhizobase/Bradyrhizobium).

In the microarray analysis under symbiotic conditions, soybean (*G. max* L. cv. Enrei) plants were inoculated with the wild-type or Δblr7984 mutant of *B. diazoefficiens* USDA110 as described above, and bacteroids were isolated from root nodules 9 weeks after the germination of plants, as described in Uchiumi *et al.* (24). The preparation of total RNA from bacteroids and a microarray analysis were performed using the same method as that for free-living cells described above.

### Real-time PCR analysis

One microliter of a glycerol stock of the wild-type or Δblr7984 mutant of *B. diazoefficiens* USDA110 was inoculated into 10 mL of YMB and pre-cultured at 28°C for 8 h. One milliliter of precultured cells was diluted in 100 mL of YMB and cultured at 28°C for 2 day. Cells were collected by centrifugation and dissolved in improved Bergersen’s synthetic medium (20) for cultivation at 28°C for 2 h. Cells were then collected by centrifugation and subjected to RNA isolation with Takara RNA iso (Takara Bio), followed by a treatment with DNase I (Takara Bio). A real-time PCR analysis of free-living cells was performed with the Smart cycler II system (Cepheid, Sunnyvale, CA, USA) and Onestep SYBR RT-PCR kit (Takara Bio). The primers used are in Supplemental Table S3. Amplification efficiency was measured using the serial dilution of total RNA. *bll7349* (*sig A*) was used as a control and the relative expression of 5 genes adjacent to the disrupted gene, *bll7981*, *bll7982*, *bll7983*, *blr7985*, and *blr7986*, to *sig A* was compared by calculating ΔCt (Ct for the gene—Ct for *sig A*). In order to compare between wild-type and Δblr7984 mutant cells, ΔΔCt (ΔCt for wild-type and −ΔCt for the mutant) was calculated and fold changes were expressed as 2^ΔΔCt^. Amplification efficiencies were almost 1 for all genes examined (Supplemental Table S4).

In the real-time PCR analysis of expression in bacteroids, bacteroids were isolated from root nodules 3, 5, and 9 weeks after the germination of plants, as described by Uchiumi *et al.* (24). Total RNA prepared with RNA iso Plus (Takara Bio) was treated with DNase I (Invitrogen) and then reverse-transcribed with SuperScript™ III (Invitrogen). The real-time PCR analysis was performed using LightCycler Nano (Roche, Basel, Switzerland) with FastStart Essential DNA Green Master (Roche). The primers used are shown in Supplemental Table S5. Absolute copy numbers were examined by using serial dilutions of the cDNA fragments of the genes with known amounts as references.

## Results

### Phenotype of the Δblr7984 mutant in the proliferation of free-living cells

The knockout mutant of the blr7984 gene was generated by inserting the Ω cassette encoding genes for resistance to spectinomycin and streptomycin into the blr7984 region of the genome of *B. diazoefficiens* USDA110. The gene organization map of blr7984 and its flanking region is shown in Supplemental Fig. S1. In order to confirm the insertion, genomes from the wild-type and Δblr7984 mutant were amplified with the primers blr7984-F and blr7984-R residing on both sides of the insertion. The length of PCR products was 2 kb longer in the Δblr7984 mutant, which corresponds to the size of the inserted Ω cassette (Supplemental Fig. S2). Furthermore, the sequence of the Ω cassette was amplified with the primers OMEGA-F and OMEGA-R from the Δblr7984 mutant only, and not from the wild-type (data not shown).

**Fig. 1:**
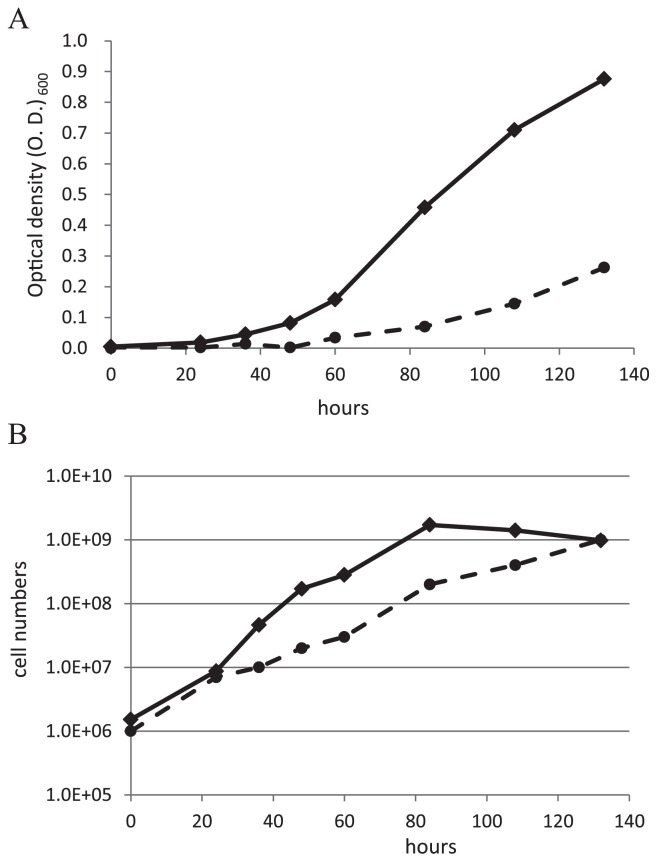
Growth rate of the blr7984 mutant. Turbidity (A) and cell numbers measured with the plate dilution method (B) in the blr7984 mutant (solid lines) and wild-type (broken lines) are shown. Data are the average of triplicate cultures.

During cultivation, we noted that the turbidity of the Δblr7984 mutant increased faster than that of the wild-type strain (Fig. 1A). We also compared the rates of increases in cell numbers using the plate dilution method (Fig. 1B). Precultured cells adjusted to 10^6^ cells mL^−1^ were grown for an additional 132 h. The cell numbers of the Δblr7984 mutant reached 10^9^ cells mL^−1^ in 80 h, whereas the same increase in cell numbers took approximately 132 h for the wild-type. Cell numbers reached a maximum at 84 h in the Δblr7984 mutant (Fig. 1B), while the turbidity of the Δblr7984 mutant continued to increase after 84 h (Fig. 1A). Since cell pellets of the Δblr7984 mutant were viscous, substances such as polysaccharides were assumed to be excreted from the cell, which explains the increase observed in turbidity even after cell proliferation had stopped.

We compared generation times calculated from the speed of increases in cell numbers measured with the plate dilution method (Table 1). Between 24 and 48 h after the start of the culture, generation times were shorter in the Δblr7984 mutant than in the wild-type strain, and the cell numbers of the mutant started to decrease after 84 h. Mutant cells appear to have grown faster and already reached the stationary phase after 84 h. In contrast, the cell numbers of the wild-type continued to increase by 132 h.

### Phenotype of the Δblr7984 mutant in symbiosis with soybean plants

**Fig. 2:**
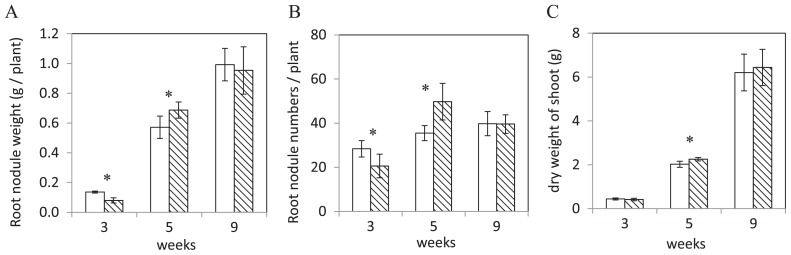
Phenotypes of the Δblr7984 mutant in symbiosis. Weights (A) and numbers (B) of visible root nodules plant^−1^ and dry weights (C) of shoots were measured 3, 5, and 9 weeks after the germination of soybean plants. White bars show root nodules derived from the wild-type and bars with diagonal lines show those from the Δblr7984 mutant. Asterisks show significant differences between the wild-type and Δblr7984 mutant (one-sided Student’s *t*-test, *n*=5, *P* <0.05).

In order to investigate the function of the blr7984 gene in symbiosis, soybean plants were inoculated with the Δblr7984 mutant and wild-type strain USDA110. As shown in Fig. 2A, the weight of visible root nodules was significantly lower, by 41%, in plants inoculated with the Δblr7984 mutant than in those inoculated with wild-type *B. diazoefficiens* 3 weeks after the germination of soybean plants, while it was 20% higher at 5 weeks (Fig. 2A). No significant difference was observed in the weight of visible nodules at 9 weeks (Fig. 2A). The number of visible root nodules with Δblr7984 was 28% lower at 3 weeks and 40% higher at 5 weeks (Fig. 2B). No significant difference was observed in the number of visible nodules at 9 weeks (Fig. 2B). The dry weight of the shoots of the soybean was significantly higher (11%) at 5 weeks with Δblr7984 than with the wild-type, whereas no significant differences were noted at 3 or 9 weeks (Fig. 2C). The nitrogen fixation activity of nodules determined as ARA activity, shoot dry weight, root fresh weight, and total nitrogen content in shoots inoculated with the Δblr7984 mutant were not significantly different from those with wild-type USDA110 at any time point examined (Supplemental Fig. S3). These results indicate that infection by the Δblr7984 mutant required more time than that by the wild-type, as demonstrated by the smaller numbers and weights of nodules at 3 weeks, but higher numbers and weights at 5 weeks, which resulted in a slight increase in the dry weight of the shoots of soybean plants.

### Comparison of gene expression in free-living cells

**Fig. 3:**
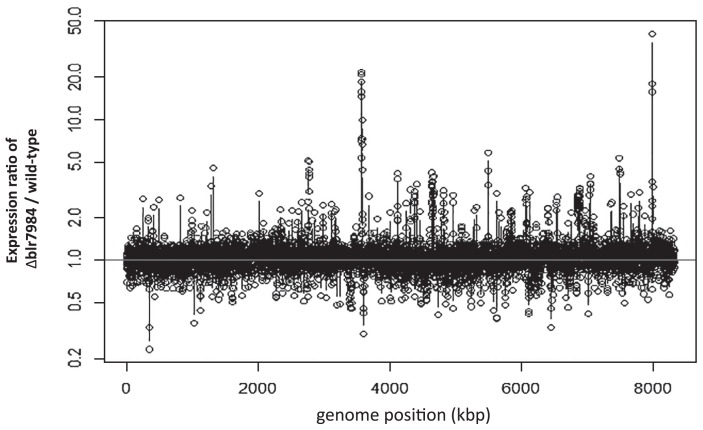
Expression ratio of Δblr7984/wild-type in free-living cells at the proliferation stage. The genome positions of clones were numbered according to the start of the replication origin as 1, which follows the Rhizobase (http://genome.microbedb.jp/rhizobase/Bradyrhizobium).

In order to elucidate the mechanisms responsible for increasing the cell division rate of the Δblr7984 mutant, gene expression profiles were compared between the Δblr7984 mutant and wild-type strain USDA110 under free-living conditions at the cell growing stage. All data of our microarray analysis are in Supplemental Table S1. As shown in Fig. 1B and Table 1, generation times were shorter in the Δblr7984 mutant than in the wild-type strain between 24 and 48 h after the start of the culture from 10^6^ cells mL^−1^; therefore, cells were subjected to a microarray analysis 36 h after the start of the culture. Fig. 3 shows the expression ratio of Δblr7984/wild-type through the whole genome region and Table 2 shows 110 genes, the expression of which was significantly higher, by more than 2.5 fold, in the Δblr7984 mutant than in the wild-type strain.

The most strongly up-regulated gene was bll7983, which resides next to disrupted blr7984, with a 48.9-fold increase, and two other genes adjacent to the disrupted gene, bll7982 and bll7981 were also strongly up-regulated, with 20.3-fold and 18.2-fold increases, respectively (Table 2 in the gray background). Several other gene clusters far from the blr7984 locus were also up-regulated in the Δblr7984 mutant (Table 2). The cluster from blr3566 to blr3577, which includes genes for the ABC transporter or PTS sugar transport system, was also strongly induced in the mutant (Table 2 labeled as B). Genes for ABC transporters from blr3566 to blr3571 were up-regulated by more than 9-fold in the mutant. The cluster from blr2763–blr2769 including *fix* genes for *cbb*3 cytochrome *c* subunits was also up-regulated, particularly the expression intensities of blr2763 encoding *fixN* and blr2764 encoding *fixO*, which were 7.1- and 5.5-fold higher than those of wild-type USDA110, respectively (Table 2 labeled as A). Another five genes from this cluster, encoding cytochrome, blr4955, blr6062, blr6128, blr7040, and blr7488, were induced in the Δblr7984 mutant. The cluster from bll6851 to blr6883 including 21 genes for flagella synthesis or movement were up-regulated 2.6- to 3.4-fold in the Δblr7984 mutant (Table 2 labeled as C). The cluster from bsr7036 to blr7039 for nitrate reductase was also induced with 2.8- to 3.6-fold increases (Table 2 labeled as D).

In contrast to the more than 100 genes induced, the expression levels of only seven genes, including a RNA polymerase sigma factor gene (bll1028) and chaperones such as the *groES* and *groELS* genes (blr5625 and blr5626), were significantly lower, by more than 2.5-fold, in Δblr7984 than in the wild-type (Supplemental Table S2).

### Comparison of gene expression in symbiosis

As described above, bll7983, next to the disrupted gene, was induced the most in free-living Δblr7984 mutant cells. bll7983 encodes glutathione *S*-transferase, which detoxifies oxidative substances with reducing power provided by glutathione. Glutathione levels in nodules are reported to be peak approximately 5 weeks after the planting of soybeans and then decrease, and this pattern corresponds to nitrogen fixation activity in nodules (3). Therefore, the detoxification of oxidative substances with glutathione is considered to be involved in the protection of nodules from senescence. We hypothesized that bll7983 encoding glutathione *S*-transferase was also strongly induced in bacteroids with the Δblr7984 mutant and may affect the expression of other genes related to senescence. In order to examine the impact of the elevated expression of bll7983 by the insertion of blr7984 on bacteroids at the senescent stage, a microarray analysis was also performed with bacteroid cells isolated from the root nodules of 9-week-old soybean plants inoculated with the Δblr7984 mutant or wild-type *B. diazoefficiens* USDA110. In contrast to the free-living status, in which various genes were up-regulated in the mutant, the expression levels of only 4 genes were significantly higher, by more than 2.5 fold, in the Δblr7984 mutant than in the wild-type in the bacteroid status (Table 3). Of these, the expression of blr7239 was only increased by 2.6-fold, whereas the other 3 genes were strongly up-regulated in the mutant, such as bll7981 by 12.8-fold, bll7982 by 4.9-fold, and bll7983 (glutathione transferase gene) by 121.4-fold, and all three genes were adjacent to disrupted blr7984 (Table 3).

### Comparison of gene expression between free-living and bacteroid cells

In our previous macroarray analysis, a genomic cluster was found to be repressed by the PBS treatment and bacteroids (16). Since blr7984 was the transcriptional repressor positioned in the cluster, it was hypothesized to be involved in gene repression in the cluster during the process of differentiation into bacteroids. Thus, the expression levels of blr7984 and its adjacent genes were compared between the free-living and bacteroid statuses. Table 4A shows comparisons of the expression of blr7984 and adjacent bll7981, bll7982, and bll7983 between the free-living and bacteroid statuses. In the wild-type, the expression of the adjacent genes, bll7981 and bll7983, was repressed in bacteroids and that of bl7982 was almost the same between free-living cells and bacteroids. Although blr7984 is annotated to be the transcriptional repressor, it is unlikely that blr7984 down-regulated the adjacent genes, bll7981 to bll7983, in bacteroids because the expression of blr7984 itself was also weaker in bacteroids than in free-living cells (Table 4A).

Table 4B–E shows a comparison of the expression of the cluster induced in free-living mutant cells (Table 2) between the bacteroid and free-living statuses. The cluster blr3566–blr3577, containing genes for ABC transporters and PTS sugar transport systems, and the cluster bll6851–blr6883, containing genes for flagellar synthesis or movement, were repressed in bacteroids both in the wild-type and Δblr7984 mutant (Table 4B and 4D). Genes for these clusters were considered to be unimportant for bacteroids. Although expression levels were similar in bacteroids between the mutant and wild-type, fold changes in the bacteroid/free-living statuses were lower in Δblr7984 than in the wild-type (Table 4B and 4D). This was attributed to the expression of these clusters in free-living Δblr7984 mutant cells being stronger than that in wild-type cells (Table 2).

The cluster of blr2763–blr2769 containing *fix* genes and bsr7036 to blr7039 containing nitrate reductase were induced in bacteroids in the wild-type strain (Table 4C and 4E). Genes for these clusters were considered to be required for symbiosis. In the Δblr7984 mutant, these clusters were not induced in bacteroids being ascribed to that expression of them were high in free living mutant cells (Table 2).

### Real-time PCR analysis

In order to confirm the reliability of the microarray analysis, the expression of 5 genes adjacent to the disrupted gene, bll7981, bll7982, bll7983, blr7985, and blr7986, was compared between free-living wild-type and Δblr7984 mutant cells using a real-time PCR analysis. As shown in Table 5, the expression of bll7983 was strongly induced in the mutant, followed by bll7982 and bll7983, while the expression of blr7985 and bll7986 was not. These results are consistent with those of the microarray analysis (Table 2 and Supplemental Table S1).

**Fig. 4:**
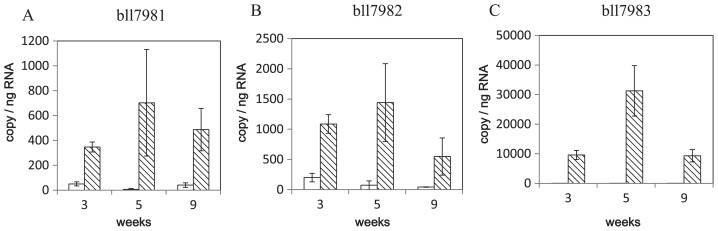
Real-time PCR analysis of the expression of bll7981, bll7982, and bll7983 in bacteroids. RNA extracted from the bacteroids of nodules 3, 5, or 9 weeks after the germination of soybean plants was subjected to a real-time PCR analysis. The means and standard deviations of three biological replicates (from different soybean plants) for bll7981 (A), bll7982 (B), and bll7983 (C) are shown. White bars are the wild-type and bars with diagonal lines are the Δblr7984 mutant.

The expression of bll7981, bll7982, and bll7983 was also compared in bacteroids from nodules 3, 5, and 9 weeks after germination using a real-time PCR analysis (Fig. 4). As in the microarray analysis, the expression of these genes was more strongly induced in bacteroids from 9-week-old nodules with the Δblr7984 mutant than in those from the wild-type. Not only 9 weeks, but also 3 and 5 weeks after germination, the expression of these three genes was induced in Δblr7984, indicating that the induction of adjacent genes by the insertion in blr7984 was not dependent on the developmental stages of nodules.

Our microarray analysis was performed with three biological replicates for the statistical analysis. In addition, good correlations were observed between the results of the microarray analysis and those of the real-time PCR analysis. Thus, our microarray analysis was considered to be reliable.

## Discussion

We previously reported that the blr7984 gene encoding a transcriptional repressor resided in the genomic region repressed in bacteroids (16). Therefore, we hypothesized that this transcription factor is involved in the regulation of gene expression during differentiation into bacteroids, generated a knockout mutant of blr7984, and observed its phenotypes.

In the Δblr7984 mutant in the free-living status, the cell division rate was increased (Fig. 1 and Table 1). In order to elucidate the mechanisms responsible for this elevation in the cell division rate, a transcriptome analysis was performed on Δblr7984 and the wild-type at cell proliferation stages. The expression levels of 110 genes were significantly higher in the Δblr7984 mutant, by more than 2.5-fold, than in the wild-type, among which three genes adjacent to blr7984 were the most strongly induced (Table 2). bll7983, next to the disrupted gene, was induced the most, by 48.9-fold. bll7983 encodes glutathione *S*-transferase, which detoxifies oxidative substances with reducing power provided by glutathione. Glutathione (GSH), a tripeptide of γ-glu-cys-gly, is a strong reductant in cells and has been reported to be involved in cell division in prokaryotes and eukaryotes. In *Sinorhizobium meliloti* and *Bradyrhizobium* sp. SEMIA 6144, a mutant defective in GSH synthesis showed slower cell division than that of the wild-type strain (7, 21). In *Arabidopsis thaliana*, a mutation in a gene for the GSH synthesis pathway caused for root meristem less phenotype, and GSH was shown to be required for the G1 to S phase transition in the cell cycle (26). A previous study also reported that GSH accumulates in the nucleus during cell proliferation in *A. thaliana* plants, triggering redox adjustments in the cytoplasm (27). In *Saccharomyces cerevisiae*, transition in the cell cycle from G1 to S requires a reductive phase, and cell cycle proteins are redox regulated at their thiol residue (2). As shown above, the redox status is known to be important for cell proliferation, both in prokaryotes and eukaryotes, and this may also be the case for *B. diazoefficiens* cells. The stronger expression of glutathione *S*-transferase encoded by bll7983 in the Δblr7984 mutant may have changed cells to the reductive phase and redox regulated important proteins for cell division, resulting in a higher cell division rate. Glutathione S-transferase is not an enzyme in glutathione synthesis, but contributes to the function of glutathione to reduce oxidative substances; therefore, it is not necessary that glutathione contents were changed by the strong expression of bll7983. The up-regulated expression of glutathione S-transferase may have accelerated the reduction of oxidative substances with electrons provided by glutathione, and the glutathione oxidized in this process was changed back to the reduced state by glutathione reductase. Among the 28 genes annotated as glutathione *S*-transferase genes of *B. diazoefficiens* USDA110 in Rhizobase, bll7983 was the only gene up-regulated more than 2.5-fold in the Δblr7984 mutant (Table 2), suggesting that glutathione *S*-transferase encoded by bll7983 is important for the regulation of the cell division rate.

In free-living Δblr7984 mutant cells, gene clusters with genomic positions far from blr7984 were also induced. blr2763 (*fixN*), blr2764 (*fixO*), and blr2765 (*fixQ*), on the *fix NOQP* operon coding for a high affinity *cbb*_3_-type oxidase complex, and blr2769 (*fixG*), blr2770 (*fixH*), and blr2771 (*fixI*), on the *fixGHIS* operon coding for a redox-coupled cation transport system essential for the biogenesis of the *cbb*_3_-type oxidase (15), were induced in Δblr7984 mutant cells (Table 2). *B. diazoefficiens* utilizes cytochrome *cbb*_3_-type oxidase to support microaerobic respiration under free-living and symbiotic conditions, and *fix NOQP* and *fixGHIS* operons are induced by low oxygen levels (1, 15), which is consistent with the expression of *fix* genes being induced in the bacteroids of the wild-type strain in Table 4C. Although Δblr7984 mutant cells were grown under aerobic conditions in our study, the higher growth rate of Δblr7984 may have required more energy than that available in the wild-type, which was compensated for by *cbb*_3_-typ oxidase. In the Δblr7984 mutant, another 5 genes for cytochromes such as blr4955, blr6062, blr6128, blr7040, and blr7488, were induced and considered to be induced to produce energy in order to support the high rate of cell division in the mutant.

The cluster from blr3566 to blr3577 was also induced in free-living Δblr7984 mutant cells (Table 2). Five genes induced in the cluster, blr3566, blr3567, blr3568, blr3570, and blr3571, encoded proteins for ABC transport systems, which uptake substances in cells using energy from ATP hydrolysis. Three genes, blr3573, blr3574, and blr3575, induced in the cluster encode proteins for the phosphotransferase (PTS) sugar system, in which they are phosphorylated and dephosphorylated in a cascading manner until the actual transporter phosphorylates the sugar during the uptake event. In addition, blr3577 encodes a homologue of the PTS-dependent dihydroxyacetone kinase subunit DhaK, which converts dihydroxyacetone to the glycolytic intermediate dihydroxyacetone phosphate using PTS as a phosphate donor (4). blr3572 is annotated as a hypothetical protein in Rhizobase, whereas an NCBI conserved domain search (http://www.ncbi.nlm.nih.gov/Structure/cdd/wrpsb.cgi) revealed that this protein has the dihydroxyacetone kinase L subunit, which uses PTS as a phosphate donor in bacteria. Thus, all genes from blr3572 to blr3577 are related to the PTS sugar transport system. This gene cluster, which is relevant to the ABC transport and PTS sugar transport systems, was considered to be induced for the uptake of substances and sugars in order to produce energy to support the higher growth rate in free-living Δblr7984 cells. In contrast, these genes were repressed in bacteroids in which cell division is repressed (Table 4B), which is consistent with gene products in this cluster supporting cell division.

The cluster from bll6851 to blr6883 including 21 genes for flagella synthesis or movement were up-regulated in the Δblr7984 mutant. A previous study reported that *B. diazoefficiens* has two sets of flagellar systems: one thick flagellum and a few thin flagella (9). All genes in the cluster from bll6851 to blr6883 induced in Δblr7984 are for the thin flagella. It was speculated that the thick flagellum is used for swimming because a mutant without the genes for the thick flagellum swam slower, while the genes for thin flagella are expressed for swarming because their expression was inducible by high viscosity (9). The cells of the free-living Δblr7984 mutant were more viscous, which may be related to the induction of genes for thin flagella.

In bacteria, the transcription of flagellin and mortality genes was organized into a hierarchy of three classes: class 1, class 2, and class 3 (10). Class 1 genes include the *filhDC* operon and gene products activate the transcription of class 2 promoters. Class 2 includes flagellar assembly and motility genes and the transcription factor required to activate the transcription of class 3 promoters. Class 3 genes are needed late in the assembly process. In *Rhizobium leguminosarum*, all of the representative genes for classes 1, 2, and 3 were expressed throughout the growth phase of free-living cells, with minimal expression at the lag and early exponential phases and maximal expression at the late exponential phase, and these genes were repressed in bacteroids in which cell growth was repressed (22). The weaker expression of the *flgH* gene for flagella synthesis in bacteroids than in free-living cells was also observed in *Mesorhizobium loti* (24). In *Rhodobacter capsulatus*, gene transcripts from classes 2 and 3 reached a maximum in the mid-log and late-log phases of growth, respectively (13). In our microarray, all 21 flagellin and mortality genes induced in the Δblr7984 mutant were classified as class 2 or 3 genes. The reason for the stronger expression of these class 2 and class 3 genes in Δblr7984 was considered to be the higher growth rate of the mutant, which reached the mid-to-late log phase at the sampling time while wild-type cells were still in the early exponential phase (Fig. 1). These genes for flagella synthesis or movement were repressed in bacteroids in which cell division is repressed (Table 4D), which is consistent with gene products in this cluster being required for cell division.

In contrast to the free-living status in which 110 genes were up-regulated more than 2.5 fold in the Δblr7984 mutant, only 4 genes were induced in the bacteroid status. Among these four genes, blr7239 was only induced by 2.6-fold, whereas the other 3 genes, bll7981 to 7983 adjacent to disrupted blr7984, were more strongly induced (Table 3). This result indicates that the disruption of the blr7984 gene directly up-regulated these three genes and that the other 107 genes, which were only up-regulated in the free-living status, but not in bacteroids, were indirectly induced to support the elevated cell division rate in the free-living status. The expression of only a small number of genes was stronger in the bacteroids of the Δblr7984 mutant than in the wild-type, which reflected the absence of changes in phenotypes in symbiosis 9 weeks after germination when bacteroids were isolated from nodules for the microarray analysis. Significant changes were observed in root nodule weight and root nodule numbers 3 and 5 weeks after germination between the wild-type and mutant (Fig. 2), suggesting the possible involvement of blr7984 genes in the early stages of symbiosis. These phenotypic changes in symbiosis in the mutant may be related to changes in the cell division rate in nodules; however, it was difficult to exactly compare these rates between the wild-type and mutant in symbiosis, unlike the free-living status.

There are two possibilities for the mechanisms underlying the up-regulation of bll7981 to bll7983 genes in the Δblr7984 mutant. One is that blr7984 is the repressor regulating these adjacent genes, and, in the Δblr7984 mutant, this repression was released. Another is that the expression of bll7981 to bll7983 was up-regulated by the promoter of the antibiotic-resistant gene inserted in the blr7984 gene locus. When the bacteroid and free-living cells of wild-type USDA110 were compared, the expression of bll7981, bll7983, and blr7984 was repressed in bacteroids (Table 4A), indicating that repression by blr7984 and the expression of adjacent genes were not linked. Thus, we were unable to confirm our hypothesis that blr7984 is the repressor of adjacent genes in the process of bacteroid differentiation. bll7981 to bll7983 may be induced in the mutant by the promoter inserted into the blr7984 locus. However, in order to confirm the latter possibility, further studies are needed in order to investigate the mode of action of the promoter inserted into the blr7984 locus. The possibility that the gene product of the inserted Ω cassette itself changed the cell division rate may be excluded because the pUTmini-Tn5 Sm/Sp vector harboring the Ω cassette was widely used for the transformation of rhizobia and changes in cell division due to the Ω cassette were not reported in such transformants (17, 28).

In summary, the Δblr7984 insertion mutant generated in this study showed the stronger expression of bll7981 to 7983 in free-living cells and bacteroids and a higher division rate of free-living cells than of wild-type cells. bll7983 encoding glutathione *S*-transferase, which was the most strongly induced in the mutant, was considered to be a possible factor elevating the cell proliferation rate in the mutant. Previous studies have suggested that redox regulation by glutathione is involved in the regulation of cell division both in prokaryotes and eukaryotes. An increase in the expression of bll7983 may have led to the redox regulation of proteins involved in cell division. Our microarray analysis detected up-regulated genes resulting from higher cell division, such as genes for energy production and uptake substances, but did not identify the proteins regulating cell division, which may be redox regulated. A proteomic analysis is needed in future studies and the mutant generated in this study, which strongly expresses bll7983, will be a useful tool for elucidating the mechanisms regulating cell division rates in *B. diazoefficiens*.

## Supplementary Information







## Figures and Tables

**Table 1:** Generation times of cells calculated from cell numbers measured with the plate dilution method (*n*=3).

Cultured time (hours)	Wild-type	Δblr7984

	(hours)	(hours)
0~24	3.4 ± 0.5	4.2 ± 0.4
24~36	8.4 ± 0.4	2.3 ± 0.1
36~48	12.0 ± 1.1	3.3 ± 1.0
48~60	16.0 ± 0.9	14.6 ± 1.0
60~84	3.6 ± 0.5	3.9 ± 1.5
84~108	12.0 ± 2.1	cell numbers decreased
108~132	9.6 ± 1.0	cell numbers decreased

**Table 2:** Genes up-regulated in Δblr7984 free-living cells 36 h after the start of the culture with fold changes in Δblr7984/wild-type >2.5.

Gene Symbol	gene_assignment	Fold change free-living (Δblr7984/wild-type)	Cluster*
bll7983	glutathione transferase	48.9	
blr3566	ABC transporter substrate-binding protein	24.3	B
blr3567	ABC transporter permease	22.2	B
bll7982	hypothetical protein	20.3	
blr3568	ABC transporter permease	19.5	B
bll7981	dehydrogenase	18.2	
blr3571	ABC transporter ATP-binding protein	17.4	B
bsr3569	hypothetical protein	16.7	B
blr3575	phosphoenolpyruvate-protein phosphotransferase	12.6	B
blr3570	ABC transporter ATP-binding protein	9.7	B
blr3572	hypothetical protein	9.0	B
blr3574	phosphocarrier protein HPr	7.6	B
blr2763	cbb3-type cytochrome c oxidase subunit I, *fixN*	7.1	A
bll5496	metabolite transport protein	6.1	
blr3576	hypothetical protein	5.9	B
blr2764	cbb3-type cytochrome c oxidase subunit II, *fixO*	5.5	A
blr7040	cytochrome C-type protein	5.3	
blr3573	PTS system mannnose-specific transporter subunit IIA	5.1	B
blr7488	cytochrome C	5.0	
blr1311	outer membrane protein	4.9	
blr2766	cbb3 oxidase subunit III, *fixP*	4.9	A
blr4638	hypothetical protein	4.8	
bll5495	hypothetical protein	4.8	
blr7489	lactoylglutathione lyase	4.8	
blr4637	HspC2 heat shock protein	4.7	
blr2767	(Fe-S)-binding protein, *fixG*	4.7	A
blr4660	ABC transporter	4.7	
blr4114	hypothetical protein	4.6	
blr4115	acetate permease	4.5	
blr4657	beta-glucosidase	4.5	
bll6888	porin	4.4	
blr7490	hypothetical protein	4.3	
blr3577	dihydroxyacetone kinase subunit DhaK	4.2	B
bsr2765	cbb3 oxidase subunit IV, *fixQ*	4.2	A
bll4640	hypothetical protein	4.2	
bll4656	hypothetical protein	4.1	
bll4412	hypothetical protein	4.1	
blr4641	hypothetical protein	3.9	
bsr4636	cation transport regulator	3.8	
bsl7992	hypothetical protein	3.8	
blr4659	PfkB family carbohydrate kinase	3.7	
blr7038	nitrate reductase catalytic subunit	3.6	D
blr1289	myosin-cross-reactive antigen/	3.6	
blr7039	periplasmic nitrate reductase small subunit	3.5	D
blr4658	glucokinase	3.5	
blr2768	FixH protein, *fixH*	3.5	A
bll2007	coproporphyrinogen III oxidase	3.5	
bll4819	hypothetical protein	3.5	
blr7037	periplasmic nitrate reductase	3.4	D
bll6881	flagellar motor switch protein FilM	3.4	C
blr6128	cytochrome c552	3.4	
blr6062	cytochrome C6	3.3	
bll4643	hypothetical protein	3.2	
bll7787	hypothetical protein	3.2	
blr4646	hypothetical protein	3.2	
blr4652	hypothetical protein	3.3	
bll6861	chemotaxis protein	3.3	C
bll4818	hypothetical protein	3.3	
bll6879	flagellar motor switch protein	3.1	C
blr6883	hypothetical protein	3.1	C
blr2762	hypothetical protein	3.1	
bll5494	hypothetical protein	3.1	
bsr4957	hypothetical protein	3.1	
blr7666	transcriptional regulator	3.0	
blr5623	hypothetical protein	3.0	
bll6869	flagellar basal body L-ring protein FlgH	3.0	C
bll6061	transcriptional regulator	3.0	
bll6867	flagellar biosynthesis protein FliP	3.0	C
blr4241	hypothetical protein	3.0	
bll6855	flagellar biosynthesis regulatory protein FlaF	3.0	C
bll6854	flagellar biosynthesis repressor FlbT	3.0	C
blr0241	1-aminocyclopropane-1-carboxylate deaminase	2.9	
bll6069	hypothetical protein	2.9	
bll6871	flagellar basal body P-ring protein FlgI	2.9	C
bll0818	hypothetical protein	2.9	
bll6882	flagellar motor protein MotA	2.9	C
bll6872	flagellar basal body P-ring biosynthesis protein FlgA	2.9	C
bll6073	poly-beta-hydroxybutyrate polymerase	2.9	
bll6857	flagellar hook-associated protein FlgK	2.8	C
bll6873	flagellar basal body rod protein FlgG	2.8	C
bll4634	hypothetical protein	2.8	
bll6876	flagellar basal-body rod protein FlgB	2.8	C
bll6856	flagellar hook-associated protein FlgL	2.8	C
blr0497	hypothetical protein	2.8	
blr2988	hypothetical protein	2.8	
bll6853	flagellar basal body rod modification protein	2.8	C
bll4816	hypothetical protein	2.8	
bsr7036	periplasmic nitrate reductase	2.8	D
bll6847	hypothetical protein	2.8	
bll2329	FAD-dependent oxidoreductase	2.8	
bll6880	hypothetical protein	2.7	C
blr6074	hypothetical protein	2.7	
bll6877	flagellar biosynthesis protein FlhB	2.7	C
bll6864	flagellar MS-ring protein FliF	2.7	C
blr4111	hypothetical protein	2.7	
blr7780	hypothetical protein	2.7	
bll3115	hypothetical protein	2.7	
blr4955	cytochrome B561	2.7	
blr4112	cation efflux system protein	2.7	
bll7990	hypothetical protein	2.7	
blr4635	molecular chaperone GroEL	2.7	
bll6874	flagellar hook-basal body protein FliE	2.6	C
bll6878	flagellar motor switch protein	2.6	C
bll6851	flagellar biosynthesis protein FlhA	2.6	C
bsr6521	hypothetical protein	2.6	
bsr3674	hypothetical protein	2.6	
bsl7372	hypothetical protein	2.6	
bll6875	flagellar basal body rod protein FlgC	2.6	C
bll7991	hypothetical protein	2.5	
blr2769	E1–E2 type cation ATPase, *fixI*	2.5	A

The adjascent genes to blr7984, bll7981, bll7982, and bll7983 were shaded in gray.

* The genes in the cluster blr2763–blr2769 containing fix genes are labeled as A, blr3566–blr3577 containing genes for ABC transporters and PTS sugar transport systems are labeled as B, bll6851–blr6883 containing genes for flagellar synteshis or movement are labeled as C and bsr7036 to blr7039 containing genes for nitrate reductase are labeled as D.

**Table 3:** Genes up-regulated in the Δblr7984 mutant in the bacteroid status with fold changes in Δblr7984/wild-type >2.5.

Gene Symbol	gene_assignment	Fold change bacteroid (Δblr7984/wild-type)
blr7239	uracil phosphoribosyltransferase	2.6
bll7981	dehydrogenase	12.8
bll7982	hypothetical protein	4.9
bll7983	glutathione transferase	121.4

**Table 4A:** Comparison of the expression of bll7981, bll7982, bll7983, and blr7984 genes between bacteroid and free-living statuses.

Gene Symbol	gene_assignment	Fold change (bacteroid/free-living)	

		wild-type	Δblr7984
bll7981	dehydrogenase	0.48	0.34
bll7982	hypothetical protein	1.94	0.47
bll7983	glutathione transferase	0.18	0.44
blr7984	transcriptional regulator	0.31	disrupted

**Table 4B:** Comparison of the expression of the cluster blr3566–blr3577 containing genes for ABC transporters and PTS sugar transport systems between bacteroid and free-living statuses.

Gene Symbol	gene_assignment	Fold change (bacteroid/free-living)	

		wild-type	Δblr7984
blr3566	ABC transporter substrate-binding protein	0.27	0.01
blr3567	ABC transporter permease	0.60	0.02
blr3568	ABC transporter permease	0.23	0.01
bsr3569	hypothetical protein	0.45	0.02
blr3570	ABC transporter ATP-binding protein	0.22	0.03
blr3571	ABC transporter ATP-binding protein	0.23	0.02
blr3572	hypothetical protein	0.19	0.02
blr3573	PTS system mannnose-specific transporter subunit IIA	0.40	0.04
blr3574	phosphocarrier protein HPr	0.19	0.03
blr3575	phosphoenolpyruvate-protein phosphotransferase	0.21	0.01
blr3576	hypothetical protein	0.17	0.03
blr3577	dihydroxyacetone kinase subunit DhaK	0.21	0.04

**Table 4C:** Comparison of the expression of the cluster blr2763–blr2769 containing *fix* genes between bacteroid and free-living statuses.

Gene Symbol	gene_assignment	Fold change (bacteroid/free-living)	

		wild-type	Δblr7984
blr2763	cbb3-type cytochrome c oxidase subunit I, *fixN*	8.58	0.86
blr2764	cbb3-type cytochrome c oxidase subunit II, *fixO*	6.66	0.67
bsr2765	cbb3 oxidase subunit IV, *fixQ*	4.23	0.58
blr2766	cbb3 oxidase subunit III, *fixP*	5.65	0.79
blr2767	(Fe-S)-binding protein, *fixG*	4.96	0.75
blr2768	FixH protein, *fixH*	4.15	0.78
blr2769	E1-E2 type cation ATPase, *fixI*	1.51	0.37

**Table 4D:** Comparison of the expression of the cluster bll6851–blr6883 containing genes for flagellar synteshis or movement between bacteroid and free-living statuses.

Gene Symbol	gene_assignment	Fold change (bacteroid/free-living)	

		wild-type	Δblr7984
bll6851	flagellar biosynthesis protein FlhA	0.07	0.02
bll6853	flagellar basal body rod modification protein	0.04	0.01
bll6854	flagellar biosynthesis repressor FlbT	0.15	0.07
bll6855	flagellar biosynthesis regulatory protein FlaF	0.04	0.02
bll6856	flagellar hook-associated protein FlgL	0.02	0.01
bll6857	flagellar hook-associated protein FlgK	0.10	0.01
bll6861	chemotaxis protein	0.11	0.03
bll6864	flagellar MS-ring protein FliF	0.06	0.02
bll6867	flagellar biosynthesis protein FliP	0.12	0.05
bll6869	flagellar basal body L-ring protein FlgH	0.10	0.03
bll6871	flagellar basal body P-ring protein FlgI	0.05	0.01
bll6872	flagellar basal body P-ring biosynthesis protein FlgA	0.05	0.02
bll6873	flagellar basal body rod protein FlgG	0.02	0.01
bll6874	flagellar hook-basal body protein FliE	0.08	0.02
bll6875	flagellar basal body rod protein FlgC	0.03	0.01
bll6876	flagellar basal-body rod protein FlgB	0.02	0.01
bll6877	flagellar biosynthesis protein FlhB	0.07	0.06
bll6878	flagellar motor switch protein	0.10	0.03
bll6879	flagellar motor switch protein	0.07	0.03
bll6880	hypothetical protein	0.04	0.03
bll6881	flagellar motor switch protein FilM	0.05	0.03
bll6882	flagellar motor protein MotA	0.07	0.01
blr6883	hypothetical protein	0.04	0.01

**Table 4E:** Comparison of the expression of the cluster bsr7036 to blr7039 containing genes for nitrate reductase between bacteroid and free-living statuses.

Gene Symbol	gene_assignment	Fold change (bacteroid/free-living)	

		wild-type	Δblr7984
bsr7036	periplasmic nitrate reductase	1.08	0.34
blr7037	periplasmic nitrate reductase	1.56	0.26
blr7038	nitrate reductase catalytic subunit	2.51	0.33

**Table 5:** Comparison of the expression of bll7981, bll7982, bll7983, blr7985, and bll7986 between Δblr7984 and the wild-type in free-living status by real-time PCR.

Gene Symbol	Fold change free-living* (Δblr7984/wild-type)
bll7981	6.68 ± 2.12
bll7982	12.41 ± 6.34
bll7983	81.58 ± 28.67
blr7985	0.51 ± 0.29
bll7986	0.20 ± 0.03

* The means ± standard deviations of three biological replicates are shown.
