# Supplementary material for: Growth Rate of and Gene Expression in Bradyrhizobium diazoefficiens USDA110 due to a Mutation in blr7984, a TetR Family Transcriptional Regulator Gene
Source: Microbes Environ. 2016 Jul 5;31(3):249–59. doi: 10.1264/jsme2.ME16056 (PMC5017801; doi:10.1264/jsme2.ME16056)
Supplement: Supplementary file 1 [file 31_249_s1.pdf]

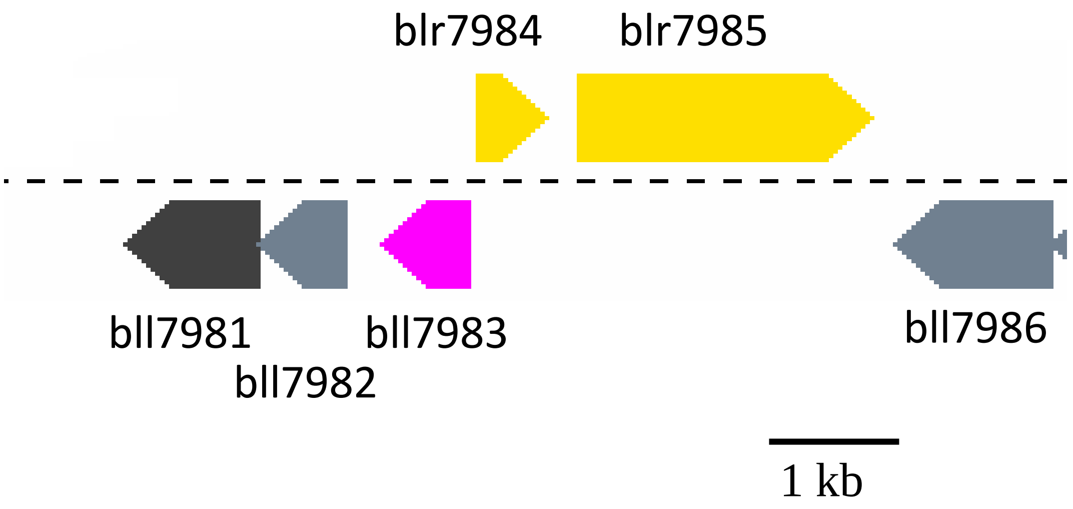


**Supplemental Fig. S1 The gene organization map of blr7984 and its franking region**

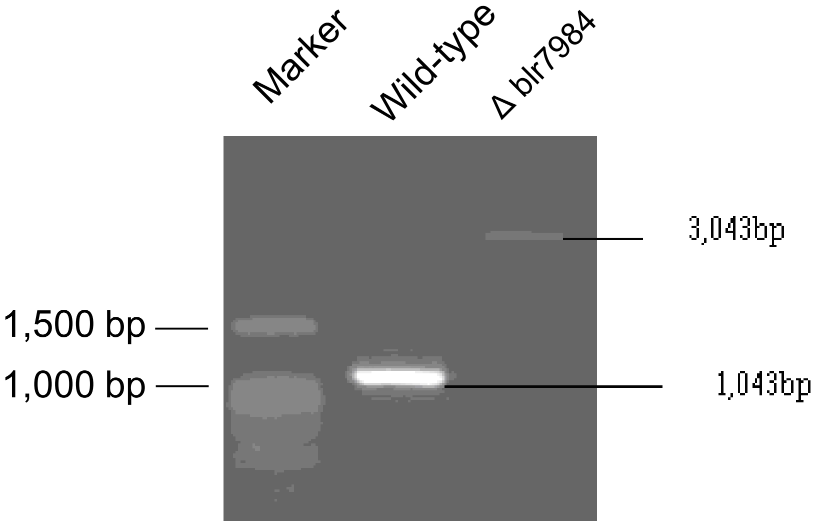


**Fig. S2 Confirmation of insertion in the *blr7984* gene.**
Genomes from wild-type and Δ blr7984 mutant were amplified with the primers blr7984-F and blr7984-R. The length of PCR products was 2 kb larger in the Δ blr7984 mutant which corresponds to the size of the inserted the Ω cassette.

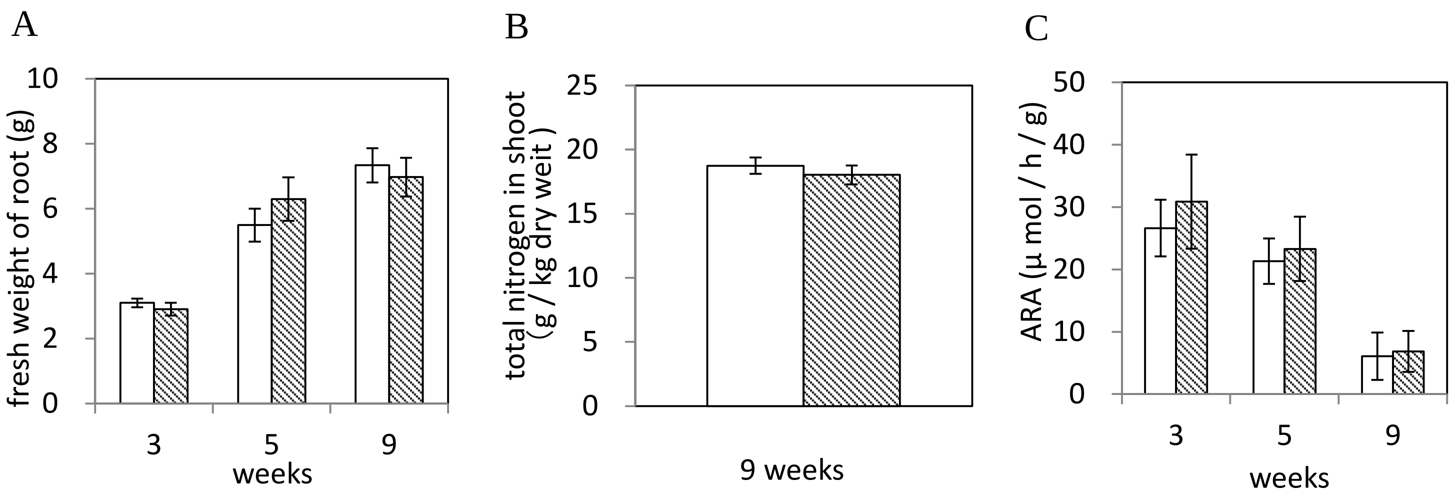


**Fig. S3 Phenotypes of the Δblr7984 mutant in symbiotic condition.**

Fresh weight or roots (A), total nitrogen content in shoot (B), and ARA activity of visible root nodules (C) of soybean plants inoculated with wild-type (white bars) or Δblr7984 rhizobium cells (bars with diagonal lines) are shown. A and C were measured at 3, 5 and 9 weeks after germination, and B was measured at 9 weeks after germination of soybean plants.
