# Supplementary material for: Growth Rate of and Gene Expression in Bradyrhizobium diazoefficiens USDA110 due to a Mutation in blr7984, a TetR Family Transcriptional Regulator Gene
Source: Microbes Environ. 2016 Jul 5;31(3):249–59. doi: 10.1264/jsme2.ME16056 (PMC5017801; doi:10.1264/jsme2.ME16056)
Supplement: Supplementary file 3 [file 31_249_s3.pdf]

Supplemental Table S2. Genes downregulated in the blr7984 cells at cellproliferation stages with fold changes Δblr7984 / wild-type < 0.25.

Gene Symbol	gene_assignment	Fold change free-living (Δblr7984 / wild-type)
bll0339	4-hydroxyphenylpyruvate dioxygenase	0.3
bsl0348	hypothetical protein	0.2
bll1028	RNA polymerase sigma factor	0.3
bll3592	hypothetical protein	0.3
blr5625	co-chaperonin GroES	0.3
blr5626	molecular chaperone GroEL	0.3
bll6449	hypothetical protein	0.3

Supplemental Table S3. Primers used for real-time PCR analysis of free-living cells.

Primers	Sequences
bll7981-F	5'-CAAGGGCATCCAGTCCAATACG-3'
bll7981-R	5'-CACGGCATATTCGTTGAAGGTG-3'
bll7982-F	5'-TCTTGCAAATTCGCATGAGCAG-3'
bll7982-R	5'-AGGTCGAAACTGTCGTCGCC-3'
bll7983-F	5'-CCGAGGTCGAGATCTATCAGAACG-3'
bll7983-R	5'-CGTTGAAGTAATGCATGCCTCTGAC-3'
blr7985-F	5'-CATCTGGTCGATGCACTTCGTC-3'
blr7985-R	5'-GAGCAGAGTCAGGAATGGATCGTAG-3'
bll7986-F	5'-GGTGAGTGCCTTGTTCCGAAAAG-3'
bll7986-R	5'-TCACCTCAGCCACGTGTCTATTG-3'
bll7349-F (sig A)	5'-CATCGCGAAGAAGTACACCAACC-3'
bll7349-R (sig A)	5'-GTGGCGTAGGTCGAGAACTTGTAG-3'

Supplemental Table S4. Amplification effieiciencies in the real-time PCR analysis of free-living cells.

Gene Symbol	Amplification effieiciency
bll7981	0.90
bll7982	0.97
bll7983	1.01
blr7985	1.05
bll7986	0.97
bll7349(sigA)	0.97

Supplemental Table S5. Primers used for real-time PCR analysis of bacteroid.

Primers	Sequences
bll7981-F	5'-CTGCGATTCTCGTCGAGTC-3'
bll7981-R	5'-TCGATCTCGTTGATCTGTGC-3'
bll7982-F	5'-CCTGCTGTACGAGATGCAAA-3'
bll7982-R	5'-TCGTCGCCATAGGGATAGAC-3'
bll7983-F	5'-TCCTCGCCAAGAACTACTCG-3'
bll7983-R	5'-GCGTCGAGATATTCGGTGAT-3'
